# Preclinical pharmacodynamics and safety profiling of AZD9773: a novel anti-TNFα polyclonal immune ovine Fab similar to D-CytoFab

**DOI:** 10.1186/cc9682

**Published:** 2011-03-11

**Authors:** P Newham, J Yates, S Das, J Kemp, J Young, P Ceuppens, F Brennan, R Knight, J Growcott

**Affiliations:** 1 AstraZeneca, Macclesfield, UK; 2 Present address: Novartis, Basel, Switzerland

## Introduction

The critical pathophysiological trigger of sepsis is thought to be a disturbance in the equilibrium between the proinflammatory response and concomitant anti-inflammatory mechanisms. Data show that the proinflammatory cytokine TNFα is a principal mediator of sepsis [1,2]. AZD9773 is a sterile lyophilised powder for solution for i.v. infusion containing ovine immune fragments (Fabs) of IgG that bind to human TNFα. We explored the PD and safety profile of AZD9773 in cynomolgus monkeys. AZD9773 PD data are compared with D-CytoFab (a similar ovine anti-TNFα IgG immune Fab product) that showed clinical benefit in a phase IIb study [3].

## Methods

AZD9773 binding and neutralisation of primate TNFα were assessed using surface plasmon resonance and TNFα-mediated cytotoxicity assay using L929 cells, respectively. AZD9773 did not show any unexpected binding to frozen primate tissue. The *in vivo *ability of either AZD9773 or D-CytoFab to suppress TNFα-mediated effects was determined by the inhibition of endotoxin-induced TNFα and IL-6 production in cynomolgus monkeys. A mathematical (PK-PD) model was constructed to describe the cytokine PD profile. Safety assessments included monitoring electrocardiogram outputs, heart rate, blood pressure and toxicology indices in cynomolgus monkeys administered with AZD9773.

## Results

There was no significant difference between AZD9773 and D-CytoFab in the binding of primate TNFα *in vitro*, and AZD9773 and D-CytoFab neutralised recombinant primate TNFα with only a twofold and 1.8-fold reduction in potency, respectively, compared with recombinant human TNFα. Both AZD9773 and D-CytoFab at equivalent doses with comparable exposure significantly suppressed endotoxin-induced IL-6 production in cynomolgus monkeys to a similar extent. PK-PD analysis revealed the effect of AZD9773 and D-CytoFab on serum TNFα and IL-6 levels and estimated model parameters were not significantly different. No toxicologically significant findings were observed in cynomolgus monkeys with AZD9773 at doses significantly higher than those currently under clinical investigation.

## Conclusions

Preclinical data indicate that AZD9773 has a good safety profile and is a well-tolerated anti-TNFα immune Fab product with PD characteristics similar to D-CytoFab.

## References

[B1] Crit Care Clin 1989 5 1

[B2] Crit Care Med 1989 17 389 10.1097/00003246-198905000-00002 2651003

[B3] Crit Care Med 2006 34 2271 10.1097/01.CCM.0000230385.82679.34 16810105

